# Cudarflavone B Provides Neuroprotection against Glutamate-Induced Mouse Hippocampal HT22 Cell Damage through the Nrf2 and PI3K/Akt Signaling Pathways

**DOI:** 10.3390/molecules190810818

**Published:** 2014-07-24

**Authors:** Dong-Sung Lee, Wonmin Ko, Dong-Cheol Kim, Youn-Chul Kim, Gil-Saeng Jeong

**Affiliations:** 1 Inha Research Institute for Medical Sciences, Inha University School of Medicine, Incheon 400-712, Korea; E-Mail: dongsunglee@inha.ac.kr; 2 College of Pharmacy, Wonkwang University, Iksan 570-749, Korea; E-Mails: rabis@wku.ac.kr (W.K.); kimman07@hanmail.net (D.-C.K.); 3 College of Pharmacy, Keimyung University, 1095 Dalgubeol-daero, Daegu 704-701, Korea

**Keywords:** cudraflavone B, glutamate, oxidative stress, neuroprotection, mouse hippocampalHT22, nuclear factor-E2-related factor 2, heme oxygenase-1

## Abstract

Oxidative cell damage contributes to neuronal degeneration in many central nervous system (CNS) diseases such as Alzheimer’s disease, Parkinson’s disease, and ischemia. Nrf2 signaling-mediated heme oxygenase (HO)-1 expression acts against oxidants that are thought to play a key role in the pathogenesis of neuronal diseases. Cudraflavone B is a prenylated flavone isolated from *C. tricuspidata* which has shown anti-proliferative activity, mouse brain monoamine oxidase (MAO) inhibitory effects, apoptotic actions in human gastric carcinoma cells and mouse melanoma cells, and hepatoprotective activity. In this study, cudraflavone B showed neuroprotective effects and reactive oxygen species (ROS) inhibition against glutamate-induced neurotoxicity by inducing the expression of HO-1 in mouse hippocampal HT22 cells. Furthermore, cudraflavone B caused the nuclear accumulation of nuclear factor-E2-related factor 2 (Nrf2) and increased the promoter activity of antioxidant response elements (ARE) in mouse hippocampal HT22 cells. In addition, we found that the Nrf2-midiated HO-1 expression by cudraflavone B is involved in the cell protective response and ROS reductions, and cudraflavone B-induced expression of HO-1 was mediated through the phosphatidylinositol 3-kinase (PI3K)/Akt pathway in HT22 cells. Our results demonstrated the potential application of naturally occurring cudraflavone B as a therapeutic agent from neurodegenerative disease.

## 1. Introduction

Neurodegeneration is the umbrella term for the progressive loss of structure or function of neurons, including death of neurons. Neuronal oxidative stress has been postulated as the underlying basis for neuronal cell death in neurodegenerative diseases such as Alzheimer’s and Parkinson’s diseases [1]. Glutamate is the main excitatory neurotransmitter in the central nervous system (CNS), but glutamate toxicity causes neuronal cell loss associated with acute insults and chronic neurodegenerative disease [2]. Glutamate toxicity also has been shown to induce neuronal cell death through both receptor-initiated excitotoxicity and non-receptor-mediated oxidative stress [3,4]. This immortalized neuronal HT22 cells, originating from mouse hippocampus, lacks functional ionotropic glutamate receptors, thus excluding excitotoxicity as a cause for glutamate triggered cell death [5]. Therefore, HT22 cells have been used as an *in vitro* model for studying the mechanism of oxidative glutamate toxicity. In this respect, naturally occurring compounds that have intrinsic anti-oxidative effects against glutamate-induced oxidative stress and which can trigger the intracellular cascade of protective pathways may offer a promising strategy for therapeutic applications. In our previous studies, certain phytochemicals were reported to protect immortalized mouse hippocampal HT22 cells against glutamate-induced oxidative damage [6,7,8].

The nuclear factor-E2-related factor 2 (Nrf2) is a transcription factor which regulates production of many antioxidant enzymes. The Nrf2 transcription factor plays an important role in the antioxidant response elements (ARE)-mediated expression of phase 2 detoxifying and in the activation of other inducible genes by various oxidative responses [9]. Exposure of cells to the naturally occurring antioxidants possessing Michael-reaction acceptors disrupts the Keap1-Nrf2 complex, allowing Nrf2 to translocate into the nucleus, where it binds to ARE and activates transcription [10]. Heme oxygenase (HO)-1 is expressed by Nrf2/ARE pathways. HO-1, also known as HSP32, belongs to the HSP family and protects mammalian cells from oxidative stress by degrading toxic heme into biliverdin, free iron (Fe^2+^), and carbon monoxide (CO). HO-1 and its enzymatic by-products appear to play an important role in regulating biological oxidative responses [11]. Moreover, Nrf2/ARE pathways and phase 2 antioxidant enzymes, including HO-1 has emerged as a therapeutic target for neuronal protections [12,13]. In this respect, the identification of constituents in natural products that have neuroprotective effects through Nrf2/ARE-mediated HO-1 expression against glutamate-induced oxidative stress would be valuable for therapeutic applications from neurodegenerative disease.

The root bark of *Cudrania tricuspidata* (Moraceae) is a traditional Chinese medicine used for the treatment of contusion, hemoptysis and lumbago [14,15,16]. Cudraflavone B, a prenylated flavone, is obtained from *C. tricuspidata* and has shown anti-proliferative activity [14], mouse brain monoamine oxidase (MAO) inhibitory effects [15], apoptotic actions in human gastric carcinoma cells and mouse melanoma cells [16,17], and hepatoprotective activity [18], but there have been no studies on the molecular targets of cudraflavone B and the mechanisms underlying its anti-neurodegenerative biological activities. In the present study, we isolated cudraflavone B and investigated its neuroprotective effects on glutamate-induced oxidative toxicity in mouse hippocampal HT22 cells through Nrf2/ARE-dependent HO-1 expression via activation of the phosphatidylinositol 3-kinase (PI3K)/AKT pathways.

## 2. Results and Discussion

### 2.1. Effects of Cudraflavone B on Glutamate-Induced Cytotoxicity and ROS Generation

**Figure 1:**
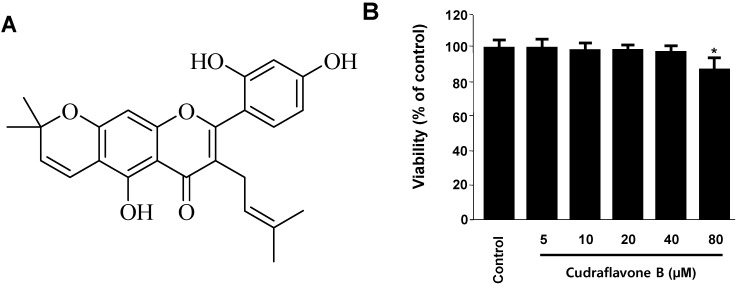
The structure of cudraflavone B (**A**) and effects of cudraflavone B on cell viability (**B**) in HT22 cells. HT22 cells were incubated for 48 h with various concentrations of cudraflavone B (5–80 μM). Data are presented as mean ± SD values of three independent experiments. * *p* < 0.05 *vs.* control.

Oxidative stress is not only an important feature of several neurodegenerative processes, but it also actively triggers intracellular signaling pathways that lead to cell death. Glutamate-induced oxidative toxicity has been observed in pathological neuronal cell damage. Therefore, therapeutic efforts aimed to mitigate the deleterious effects of ROS or prevent their formation may prove beneficial to neuronal cells [1]. To determine the cytotoxic potential of cudraflavone B (Figure 1A), its effects on viability of HT22 cells (Figure 1B) was evaluated. A concentration of 40 μM revealed no cytotoxic effects using the MTT assay. However, a higher concentration (80 μM) showed a slightly reduced viability of these cells (Figure 1B).

**Figure 2:**
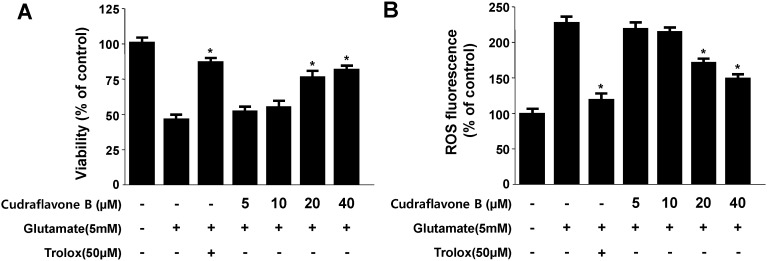
Effects of cudraflavone B on glutamate-induced oxidative neurotoxicity (**A**) and ROS generation (**B**) in HT22 cells.HT22 cells were pre-treated with cudraflavone B for 12 h and then incubated for 12 h with glutamate (5 mM). Exposure of HT22 cells to glutamate increased ROS production. Data are presented as mean ± SD values of three independent experiments. Trolox (50 μM) was used as the positive control. * *p* < 0.05 *vs.* glutamate.

Glutamate induces oxidative stress by inhibiting the cellular uptake of cystine via the cystine/glutamate transport system, Xc^−^, leading to depletion of glutathione, increased ROS production, and elevated Ca^2+^ levels [3,4]. In this study, we examined the protective effects of cudraflavone B against glutamate-induced cytotoxicity in HT22 cells. Treatment with glutamate increased HT22 cell death to 48% ± 2.2% compared to the untreated cells, and at the non-cytotoxic concentrations, cudraflavone B (20 and 40 μM) increased viability dose-dependently (Figure 2A). Cudraflavone B showed potent protective effects on glutamate-induced cytotoxicity exhibiting an EC_50_ value of 23.1 ± 3.7 μM. Glutamate also doubled ROS production, and cudraflavone B effectively suppressed this induction and exhibited the EC_50_ value of 19.4 ± 4.1 μM (Figure 2B). Trolox (6-hydroxy-2,5,7,8-tetramethylchroman-2-carboxylic acid), well known for its anti-oxidative efficiency, was used as positive control, and showed a significantly cytoprotective effect and ROS scavenging activity at a concentration of 50 μM (Figure 2A,B).

### 2.2. Effects of Cudraflavone B on HO-1 mRNA and Protein Expression in HT22 Cells

**Figure 3:**
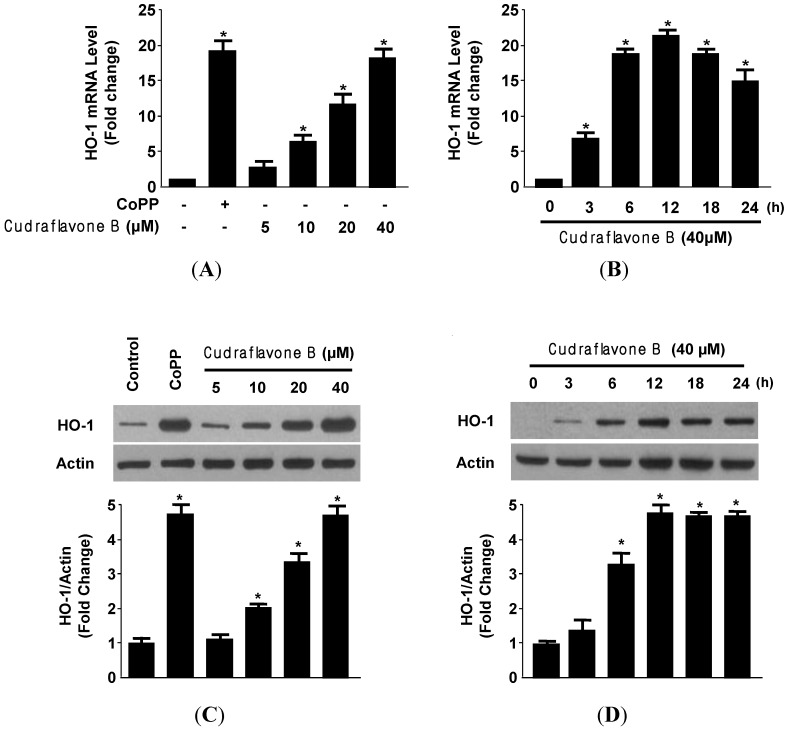
Effects of cudraflavone B on HO-1 mRNA (**A**, **B**) and protein (**C**, **D**) expression in HT22 cells. HT22 cells were incubated with indicated concentrations of cudraflavone B for 12 h (**A**, **C**) and cudraflavone B (40 μM) for the indicated times (**B**, **D**). Data are presented as the mean ± SD values of three independent experiments. CoPP (20 μM) was used as the positive control. * *p* < 0.05 *vs.* control.

It is well known that the cytoprotective properties of antioxidants have been partially attributed to their abilities to induce cytoprotective enzymes. Although HO-1 does not directly catalyze an antioxidant reaction, its induction is generally considered an adaptive cytoprotective response against the toxicity of oxidative stress [19]. Therefore, we examined whether non-cytotoxic concentrations (5–40 μM) of cudraflavone B affected HO-1 mRNA and protein expression by treating the cells with cudraflavone B for 12 h. As shown in Figure 3, we found that cudraflavone B significantly increased HO-1 mRNA and protein levels in a concentration-dependent manner, with a maximal value observed at 40 μM (Figure 3A,C).

The HO-1 inducer CoPP, used as a positive control, increased HO-1 expression at a concentration of 20 μM. When cudraflavone B was used at a concentration of 40 μM, HO-1 mRNA and protein expression were evident at 6 h and peaks at around 12 h (Figure 3B,D). These result suggested that the neuroprotective effect of cudraflavone B against glutamate-induced cell death partially attributes to HO-1 in HT22 cells. HO-1 is highly inducible and expressed in neuronal cells, and also has cytoprotective effects against glutamate-induced toxicity in HT22 cells [20].

### 2.3. Effects of Cudraflavone B on Nrf2 Nuclear Translocation and Nrf2-mediated HO-1 Expression in HT22 Cells

**Figure 4:**
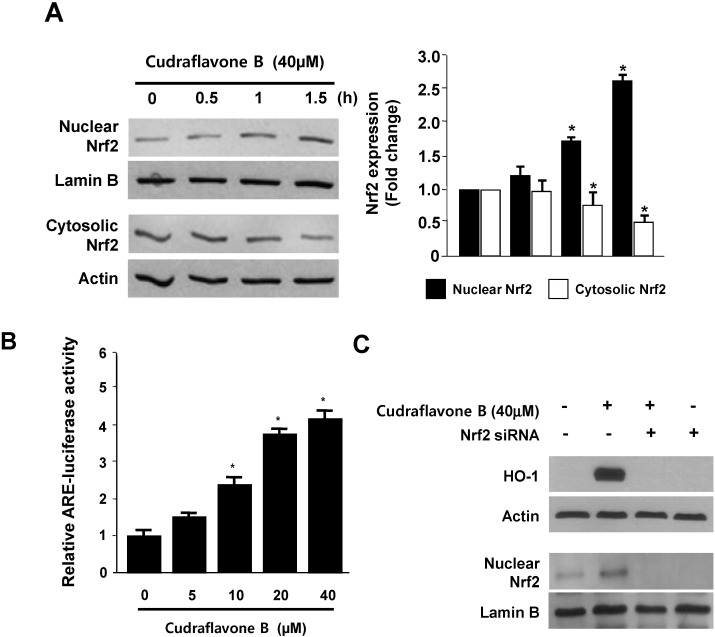
Effects of cudraflavone B on the nuclear translocation of Nrf2 (**A**); ARE activation (**B**); and Nrf2-mediated HO-1 expression (**C**) in HT22 cells. HT22 cells were treated with cudraflavone B (40 μM) for 0.5, 1, and 1.5 h (**A**); Quiescent cells transiently transfected with ARE-luciferase or control vector were incubated for 1 h with indicated concentrations of cudraflavone B in the presence of 5% FBS (**B**); HT22 cells were transiently transfected with Nrf2 siRNA and then treated with cudraflavone B (40 μM) for 12 h (HO-1) or 1.5 h (Nuclear Nrf2) (**C**). Data are presented as the mean value ± SD values of three independent experiments. * *p* < 0.05 *vs.* control.

When cells are subjected to a variety of oxidative stresses, they typically respond by inducing a coordinated expression of genes encoding the set of phase II detoxifying enzymes, principally involved in activation of the transcription factor including Nrf2 [21]. Previous studies have also described the protective effects of HO-1 expression by Nrf2 activation, especially reduction of oxidative stress in various models of neurodegenerative disorder [22]. Therefore, we investigated whether treatment with cudraflavone B induces the translocation of Nrf2 to the nuclei in HT22 cells. Cells were treated with 40 μM cudraflavone B for 0.5, 1, and 1.5 h, and the level of Nrf2 protein was then determined by western blotting, which indicated that the nuclear fraction of cudraflavone B-treated HT22 cells showed a gradual increase in Nrf2 levels, whereas a concomitant decrease was observed in the cytoplasmic fractions (Figure 4A). In addition, HT22 cells transiently transfected with the ARE-luciferase plasmid were exposed to cudraflavone B, and changes in luciferase activity were used as a measure of ARE activation. The reporter assay showed that cudraflavone B dose-dependently increases ARE-driven luciferase activity (Figure 4B), and this ARE activation strongly correlates with the increase in HO-1 expression (Figure 3). Furthermore, the role of Nrf2 in HO-1 expression by cudraflavone B was studied using siRNA against Nrf2. HT22 cells were transiently transfected with siRNA Nrf2 and then were treated with 40 μM cudraflavone B for 12 h (HO-1) or 1.5 h (Nuclear Nrf2). As shown in Figure 4C, transient transfection with Nrf2 siRNA completely abolishes HO-1 expression or nuclear translocation of Nrf2 by cudraflavone B. The previous reports suggested that phytochemicals can regulate the Nrf2 translocation by directly binding to Keap1, and these results in the induction of some cytoprotective proteins, including HO-1 [10]. These our results suggest that cudraflavone B-induced HO-1 expression occurs through the Nrf2/ARE signaling pathway in HT22 cells.

### 2.4. Effects of Cudraflavone B on Cell Viability and in ROS Generation via Nrf2-mediated HO-1 Expression

**Figure 5:**
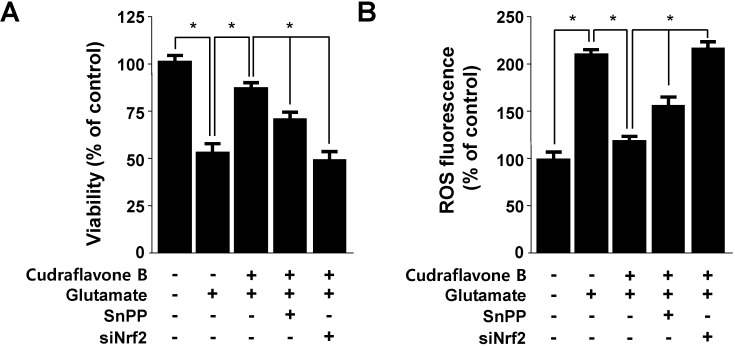
Effects of cudraflavone B-induced Nrf2-mediated HO-1 expression on glutamate-induced oxidative neurotoxicity (**A**) and ROS generation (**B**) in HT22 cells. HT22 cells were treated with 40 μM of cudraflavone B in the presence or absence of 50 μM SnPP IX and Nrf2 siRNA, after then exposed to glutamate (5 mM) for 12 h (**A**, **B**). Data are presented as the mean ± SD values of three independent experiments. Trolox was used as the positive control. * *p* < 0.05.

In our recent report, the expression of HO-1 was considered to be an adaptive and protective response against oxidative insult in a wide variety of cells, including neuronal HT22 cells [6,7]. Next, we examined whether the induction of HO-1 expression by cudraflavone B is involved in the protective effect and ROS inhibition in HT22 cells. HT22 cells were co-treated with 40 μM of cudraflavone B for 12 h in the absence or presence of SnPP IX, a competitive inhibitor of HO activity. SnPP IX significantly suppressed cudraflavone B-mediated cell protection and ROS deduction (Figure 5). These results suggest that HO-1 expression by cudraflavone B is related to the protective effect and ROS inhibition in HT22 cells. In this result, SnPP partially reversed the ability of cudraflavone B to suppress glutamate-induced cytotoxicity and ROS generation (Figure 5). Nrf2 plays a critical part in basal activity and coordinated induction of genes encoding numerous antioxidant and phase II detoxifying enzymes and related proteins, such as HO-1, glutathione (GSH), catalase, superoxide dismutase (SOD), glutathione-S-transferase (GST), γ-glutamyl cysteine ligase (GCL), NAD(P)H:quinone oxidoreductase-1 (NQO1), glutathione peroxidase, glutathione reductase (GR), and so on. These phase II detoxifying enzymes and related proteins play important roles in protecting cells from free radical and oxidative stress imposed by reactive oxygen species [23]. In this study, we provided evidence to support the view that HO-1 expression, one of the key phase II detoxifying enzymes, through Nrf2 signaling pathways plays a key role in mediating the neuro-protective effects of cudraflavone B. In our Figure 5, SnPP treatment partly attenuated the ability of cudraflavone B to suppress t-BHP-induced ROS generation, meaning that the other phase II detoxifying enzymes as well as HO-1 may be involved in the mechanism of the cytoprotective effects by cudraflavone B. Therefore, we also experimented that the role of Nrf2 translocation by cudraflavone B on the protective effect and ROS inhibition was studied using siRNA against Nrf2. HT22 cells were transiently transfected with siRNA Nrf2, and then were treated with 40 μM cudraflavone B followed by glutamate stimulation. As shown in Figure 5, transient transfection with siRNA Nrf2 reversed the reduced effects of cudraflavone B on the cell protection and ROS inhibition. These results strongly indicate that in our experimental setting, the observed cytoprotective effects of cudraflavone B were mediated through the Nrf2-midiated HO-1 expression.

**Figure 6:**
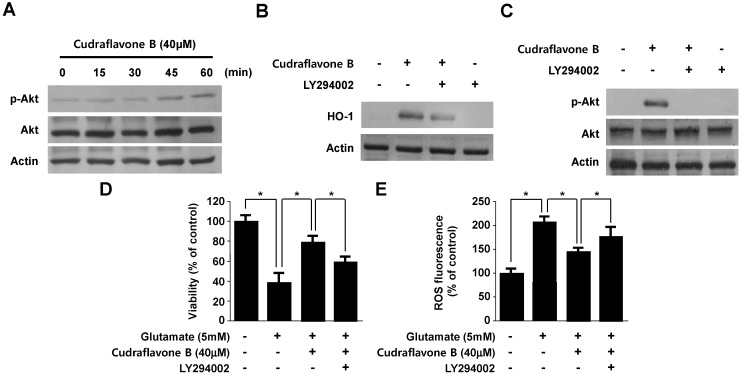
Effects of cudraflavone B-induced HO-1 expression through the PI3K/AKT cascade in HT22 cells. HT22 cells were treated with cudraflavone B (40 μM) for the indicated times (**A**); HT22 cells were pre-incubated with or without 10 μM LY294002 for 1 h and then incubated in the absence or presence of 40 μM of cudraflavone B for 12 h (**B**) or 30 min (**C**). HT22 cells untreated or treated with cudraflavone B (40 μM) in the presence or absence of LY294002 (10 μM) for 12 h were exposed to 5 mM glutamate for 12 h (**D**, **E**). Data are presented as the mean ± SD values of three independent experiments. * *p* < 0.05.

### 2.5. Involvement of the PI3K/Akt Pathway in Cudraflavone B-induced HO-1 Expression

Phosphatidylinositol 3-kinase (PI3K)/Akt signaling pathways are activated by the various oxidative responses. The Nrf2/ARE signaling is mainly regulates the Nrf2 nuclear translocation by the upstream signaling molecules, including PI3K/Akt pathways. Therefore, PI3K/Akt pathways are a pivotal regulation factor of Nrf2-induced signaling transduction [24]. PI3K/Akt signaling pathways are also considered to the expression HO-1 induced by various phytochemicals [25,26]. Therefore, we tested whether cudraflavone B-induced expression of HO-1 occurs through the PI3K pathway. To correlate the activation of Akt with the induction of HO-1 by cudraflavone B, we investigated Akt phosphorylation in HT22 cells using an anti-phospho-Akt antibody. The phosphorylation of Akt was observed from 10 to 60 min (Figure 6A). Moreover, Figure 6B showed that pretreatment with 10 μM LY294002 (a specific inhibitor of PI3K) significantly attenuated HO-1 expression induced by cudraflavone B in HT22 cells. Pretreatment of the cells with LY294002 attenuated the phosphorylation of Akt in response to cudraflavone B (Figure 6C). Furthermore, the PI3K pathway inhibitor (LY294002) abolished cudraflavone B-induced cytoprotection and ROS reduction (Figure 6D,E). Therefore, these results suggested that cudraflavone B-induced expression of HO-1 was mediated through the PI3K/Akt pathway in HT22 cells. Specific protein kinase inhibitors attenuated the activations of the PI3K/Akt pathways and cytoprotective effects by cudraflavone B in HT22 cells. Choi and Kim [25] reported that brazilin increased HO-1 expression through the activation of PI3K/Akt cascade pathways. Our results also indicate that PI3K/Akt cascade pathways, which are activated by cudraflavone B, participate in an early stage of HO-1expression in HT22 cells. However, further studies are extensively ongoing to define the exact role of the PI3K/Akt cascade and MAPK pathways in HO-1 expression.

## 3. Experimental

### 3.1. Chemicals and Reagents

Dulbecco’s modified Eagle’s medium (DMEM), fetal bovine serum (FBS), and other tissue culture reagents were purchased from Gibco BRL Co (Carlsbad, CA, USA). Cobalt protoporphyrin (CoPP; HO-1 inducer), an inducer of HO-1, and tin protoporphyrin IX (SnPP IX; HO inhibitor), an inhibitor of HO activity, were obtained from Porphyrin Products (Logan, UT, USA). LY294002 was obtained from Calbiochem (Darmstadt, Germany). Lipofectamine 2000^TM^ was purchased from Invitrogen Life Technologies (Grand Island, NY, USA). All other chemicals were obtained from Sigma Chemical Co. (St. Louis, MO, USA) unless otherwise stated. Small interfering RNA (siRNA) for Nrf2 [27] and primary antibodies, including mouse/goat/rabbit anti-HO-1, Nrf-2, p-Akt, Akt, and Actin, and secondary antibodies [6,7,8,28] were purchased from Santa Cruz Biotechnology (Santa Cruz, CA, USA).

### 3.2. Plant Materials and Isolation of Cudraflavone B

Cudraflavone B was isolated from *C. tricuspidata* as described previously [29]. The root barks of *Cudrania tricuspidata* were purchased in June 2004 at Kumsan Crude drug market, Chungnam Province, Korea, and identified by Dr. Kyu-Kwan Jang, Botanical Garden, Wonkwang University. A voucher specimen (No. WP 527) was deposited at the Herbarium of the College of Pharmacy, Wonkwang University (Iksan, Korea). The dried and pulverized root barks of *C. tricuspidata* (4 kg) were extracted twice with hot MeOH (20 L) for 2 h. The concentrated MeOH extract (168 g) was partitioned between equal volumes of *n*-hexane and 60% aqueous MeOH, and the aqueous MeOH layer extracted subsequently with CHCl_3_. Finally, the 60% aqueous MeOH mixtures was evaporated *in vacuo* and partitioned between *n*-BuOH and H_2_O. A portion (35 g) of hepatoprotective CHCl_3_-soluble fraction (53.3 g) was chromatographed on silica gel column (6.5 × 60 cm) using CHCl_3_/MeOH (70:1, 50:1, 30:1, 15:1, 9:1, 5:1, 1:1, MeOH, each 2 L) as eluent to obtain six fractions (Fr. 1—6). Fr. 1 (2.3 g) was chromatographed on a RP C-18 column (3 × 35 cm) eluted with 90% aqueous MeOH to yield four fractions (Fr. A—D). Fr. B (330 mg) was chromatographed on a silica column (3.0 × 30 cm, eluent; CH_2_Cl_2_/MeOH) to yield three subfractions (Fr. B1–B3). Fr. B2 (150 mg) was subjected to RP C-18 column (2.0 × 30 cm) chromatography (eluent; 65% aqueous MeOH) to obtain cudraflavone B (28.0 mg). Cudraflavone B was obtained as yellow powders, showed a [M + H]^+^ peak at *m/z* 437 in the ESI-MS, corresponding to a molecular formula of C_26_H_28_O_6_. The purity and structure of cudraflavone B was checked by ^1^H, ^13^C, HSQC, COSY and HMBC NMR spectra, and its spectra showed highly pure signals without any other impurities (Supplementary Figure S1–S5). The spectral data were identical with those reported in the literature [30]. For each experiment, cudraflavone B was dissolved in dimethyl sulfoxide and added to medium at a final concentration of 0.05%. Serum-free medium was used as a vehicle control. Preliminary studies indicated that the solvent had no effect on cell viability at the concentration used. Cudraflavone B (>93.25%) was deposited at the Standardized Material Bank for New Botanical Drugs, Wonkwang University (Korea).

### 3.3. Cell Culture and Viability Assay

Mouse hippocampal HT22 cells were received from Dr. Inhee-Mook (Seoul National University, Korea). The cells were maintained at 5 × 10^6^ cells/dish in 100-mm dishes in DMEM supplemented with 10% heat-inactivated FBS, penicillin G (100 units/mL), streptomycin (100 mg/mL), and l-glutamine (2 mM), and incubated at 37 °C in a humidified atmosphere containing 5% CO_2_ and 95% air. For determination of cell viability, cells (2 × 10^4^ cells/well in 96-well plates) were incubated with 3-(4,5-dimethylthiazol-2-yl)-2,5-diphenyltetrazolium bromide (MTT) at a final concentration of 0.5 mg/mL for 4 h, and the formazan formed was dissolved in acidic 2-propanol. Optical density was measured at 590 nm with a microplate reader (Bio-Rad, Hercules, CA, USA). The optical density of the formazan formed in control (untreated) cells was considered to represent 100% viability.

### 3.4. ROS Measurement

For measurement of ROS, HT22 cells (2.5 × 10^4^ cells/well in 24-well plates) were treated with 5 mM glutamate in the presence or absence of cudraflavone B or SnPP IX (HO inhibitor) and incubated for 8 h. After washing with phosphate-buffered saline (PBS), the cells were stained with 10 μM 2',7'-dichlorofluorescein diacetate (DCFDA) in Hank’s balanced salt solution for 30 min in the dark. The cells were then washed twice with PBS and extracted with 1% Triton X-100 in PBS for 10 min at 37 °C. Fluorescence was recorded at an excitation wavelength of 490 nm and an emission wavelength of 525 nm (Spectramax Gemini XS; Molecular Devices, Sunnyvale, CA, USA). Cells were immediately observed under a laser-scanning confocal microscope (TCS SP2, Leica, Wetzlar, Germany). DCF fluorescence was excited at 488 nm with an argon laser, and the evoked emission was filtered with a 515-nm long pass filter.

### 3.5. Preparation of Nuclear and Cytosolic Fractions

Cells were homogenized (1:20, w:v) in PER-Mammalian Protein Extraction buffer (Pierce Biotechnology, Rockford, IL, USA) containing freshly added protease inhibitor cocktail I (EMD Biosciences, San Diego, CA, USA) and 1 mM phenylmethylsulfonyl fluoride. The cytosolic fraction of the cell was prepared by centrifugation at 15,000 *×g* for 10 min at 4 °C. Nuclear and cytoplasmic extracts of HT22 cells were prepared using NE-PER nuclear and cytoplasmic extraction reagents (Pierce Biotechnology), respectively.

### 3.6. Western Blot Analysis

Cells were harvested and pelleted by centrifugation at 200 ×*g* for 3 min. Subsequently, the cells were washed with PBS and lysed in 20 mM Tris-HCl buffer (pH 7.4) containing a protease inhibitor mixture (0.1 mM phenylmethylsulfonyl fluoride, 5 mg/mL aprotinin, 5 mg/mL pepstatin A, and 1 mg/mL chymostatin). The protein concentration was determined using the Lowry Protein Assay Kit (P5626; Sigma Chemical Co.). An equal amount of protein from each sample was resolved using 12% sodium dodecyl sulfate-polyacrylamide gel electrophoresis and then electrophoretically transferred onto a Hybond-enhanced chemiluminescence nitrocellulose membrane (Bio-Rad). The membrane was blocked with 5% skimmed milk and incubated with anti-HO-1, anti-Nrf2, anti-Akt, anti-phospho-Akt, or anti-actin antibodies (all of which were used at a 1:1000 dilution and purchased from Santa Cruz Biotechnology) at 4 °C overnight. The immunoreactive bands were visualized using a horseradish peroxidase-conjugated secondary antibody (1:1000 dilution; Santa Cruz Biotechnology) followed by enhanced chemiluminescence detection (Amersham Pharmacia Biotech, Piscataway, NJ, USA) and quantified using an image analysis program (Image Gauge v3.12 software; Fujifilm, Tokyo, Japan).

### 3.7. Real-Time PCR

Total RNA was isolated from the cells by using Trizol (Invitrogen, Carlsbad, CA, USA), in accordance with the manufacturer’s recommendations, and quantified spectrophotometrically (at 260 nm). Total RNA (1 μg) was reverse-transcribed using the High Capacity RNA-to-cDNA kit (Applied Biosystems, Carlsbad, CA, USA). The cDNA was then amplified using the SYBR Premix Ex Taq kit (TaKaRa Bio Inc., Shiga, Japan) by using a StepOnePlus Real-Time PCR system (Applied Biosystems). Briefly, each 20 μL of reaction volume contained 10 μL of SYBR Green PCR Master Mix, 0.8 μM of each primer, and diethyl pyrocarbonate (DEPC)-treated water. The primer sequences were designed using PrimerQuest (Integrated DNA Technologies, Cambridge, MA, USA). The primer sequences were as follows: HO-1, forward 5'-CTCTTGGCTGGCTTCCTT-3', reverse 5'-GGCTCCTTCCTCCTTTCC-3', and glyceraldehyde 3-phosphate dehydrogenase (GAPDH), forward 5'-ACTTTGGTATCGTGGAAGGACT-3', reverse 5'-GTAGAGGCAGGGATGATGTTCT-3. The optimal conditions for PCR amplification of the cDNA were established by following the manufacturer’s instructions. The data were analyzed using StepOne software (Applied Biosystems), and the cycle number at the linear amplification threshold (Ct) values for the endogenous control gene (GAPDH) and the target gene were recorded. Relative gene expression (target gene expression normalized to the expression of the endogenous control gene) was calculated using the comparative Ct method (2^−ΔΔCt^).

### 3.8. Transfection

The cells were transiently transfected with Nrf2 siRNA for 6 h by using LipofectAMINE 2000^TM^ (Invitrogen), according to the manufacturer’s protocol, and recovered in fresh media containing 10% FBS for 24 h.

### 3.9. Plasmids, Transfections, and Luciferase Assays

To construct the ARE-luciferase vector, tandem repeats of double-stranded oligonucleotides spanning the Nrf2 binding site 5'-TGACTCAGCA-3' were introduced into the restriction sites of the pGL2 promoter plasmid (Promega, Madison, WI, USA). All transfection experiments were performed using Lipofectamine reagent (Invitrogen) according to the manufacturer’s instructions. For luciferase assays, the cell lysate was first mixed with the luciferase substrate solution (Promega), and luciferase activity was measured using a luminometer. For each experiment, luciferase activity was determined in triplicate and normalized for each sample using β-galactosidase activity.

### 3.10. Statistical Analysis

Data were expressed as the mean ± SD of at least three independent experiments. To compare three or more groups, one-way analysis of variance followed by the Newman-Keuls *post hoc* test was used. Statistical analysis was performed with GraphPad Prism software, version 3.03 (GraphPad Software Inc., San Diego, CA, USA).

## 4. Conclusions

The results of the present study suggest that cudraflavone B from *Cudrania tricuspidata* effectively prevents glutamate-induced oxidative damage, and HO-1 induction via PI3K/Akt and Nrf2/ARE pathways appears to play a key role in the protection of HT22 cells. This study provides evidence that isolated cudraflavone B can exert a neuroprotective effect via activation of the Nrf2-mediated HO-1 expression. To our knowledge, the present study is the first to show that the natural compound, cudraflavone B activates Nrf2-mediated HO-1 signaling in HT22 cells and exerts an anti-oxidative defense mechanism against glutamate-induced neurotoxicity. Therefore, this study implies that the Nrf2/HO-1 pathway represents a pharmacological target and that cudraflavone B might be a candidate for the prevention of neurodegeneration.

## Supplementary Materials

Supplementary materials can be accessed at: http://www.mdpi.com/1420-3049/19/8/10818/s1.

## Supplementary Files

Supplementary File 1
