# Supplementary material for: Cudarflavone B Provides Neuroprotection against Glutamate-Induced Mouse Hippocampal HT22 Cell Damage through the Nrf2 and PI3K/Akt Signaling Pathways
Source: Molecules. 2014 Jul 24;19(8):10818–31. doi: 10.3390/molecules190810818 (PMC6271666; doi:10.3390/molecules190810818)

Supplementary Information

Table of Contents

Figure S1. ^{1}H-NMR spectrum of cudarflavone B
Figure S2. ^{13}C-NMR spectrum of cudarflavone B
Figure S3. HSQC data of cudarflavone B
Figure S4. COSY data of cudarflavone B
Figure S5. HMBC data of cudarflavone B

Figure S1. ^{1}H-NMR spectrum of cudarflavone B.

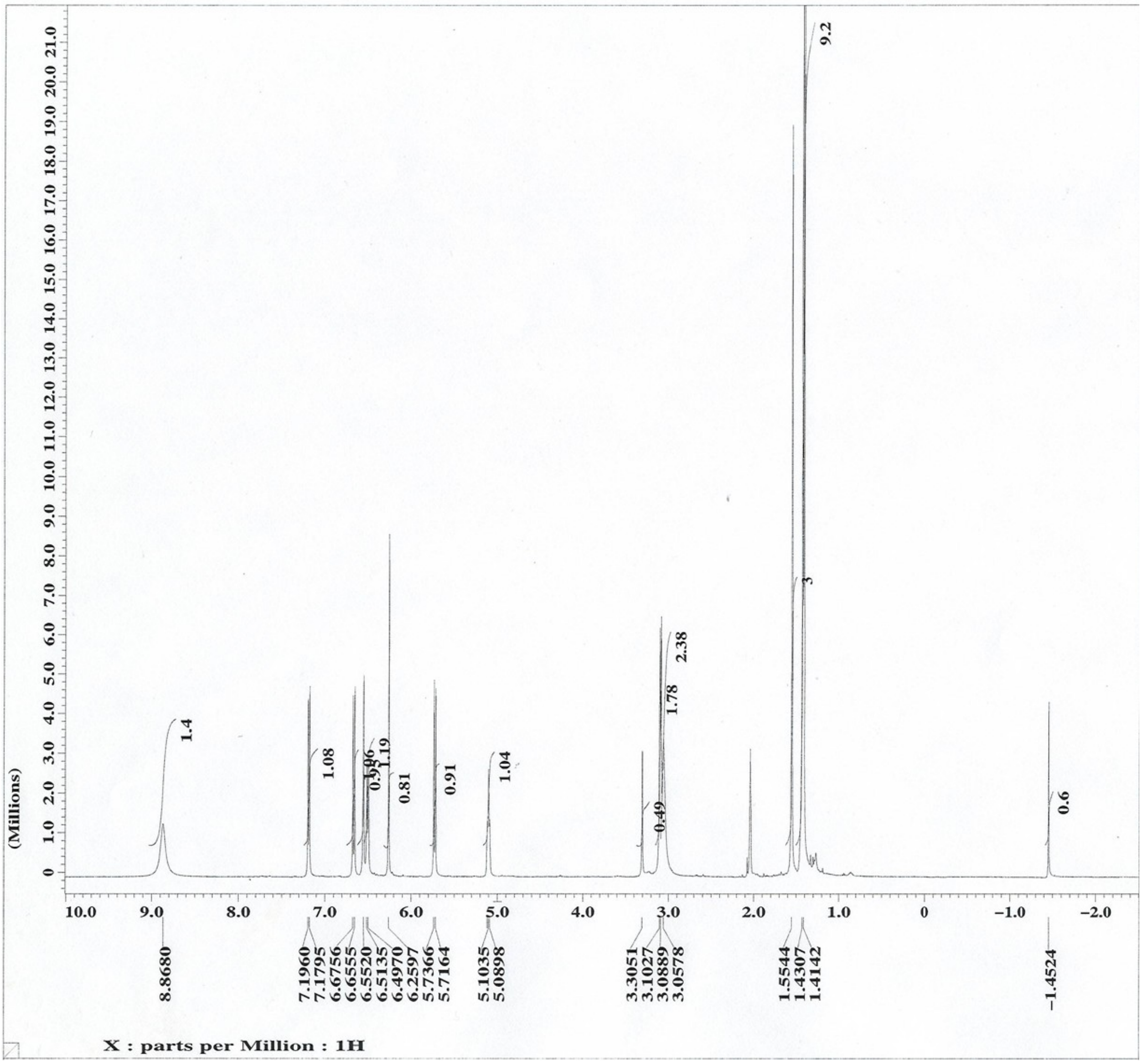

Figure S2. ^{13}C-NMR spectrum of cudarflavone B.

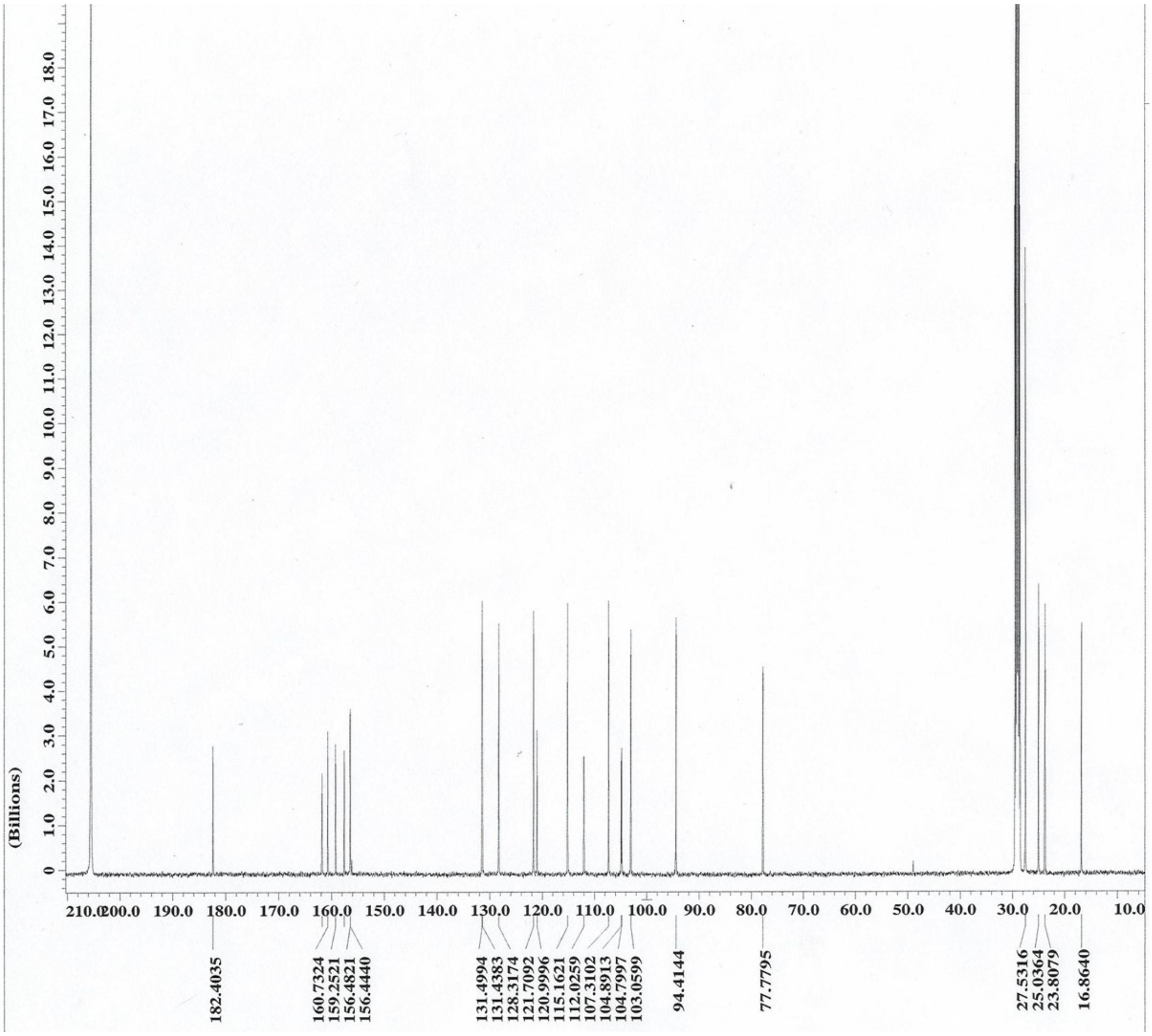

Figure S3. HSQC data of cudarflavone B.

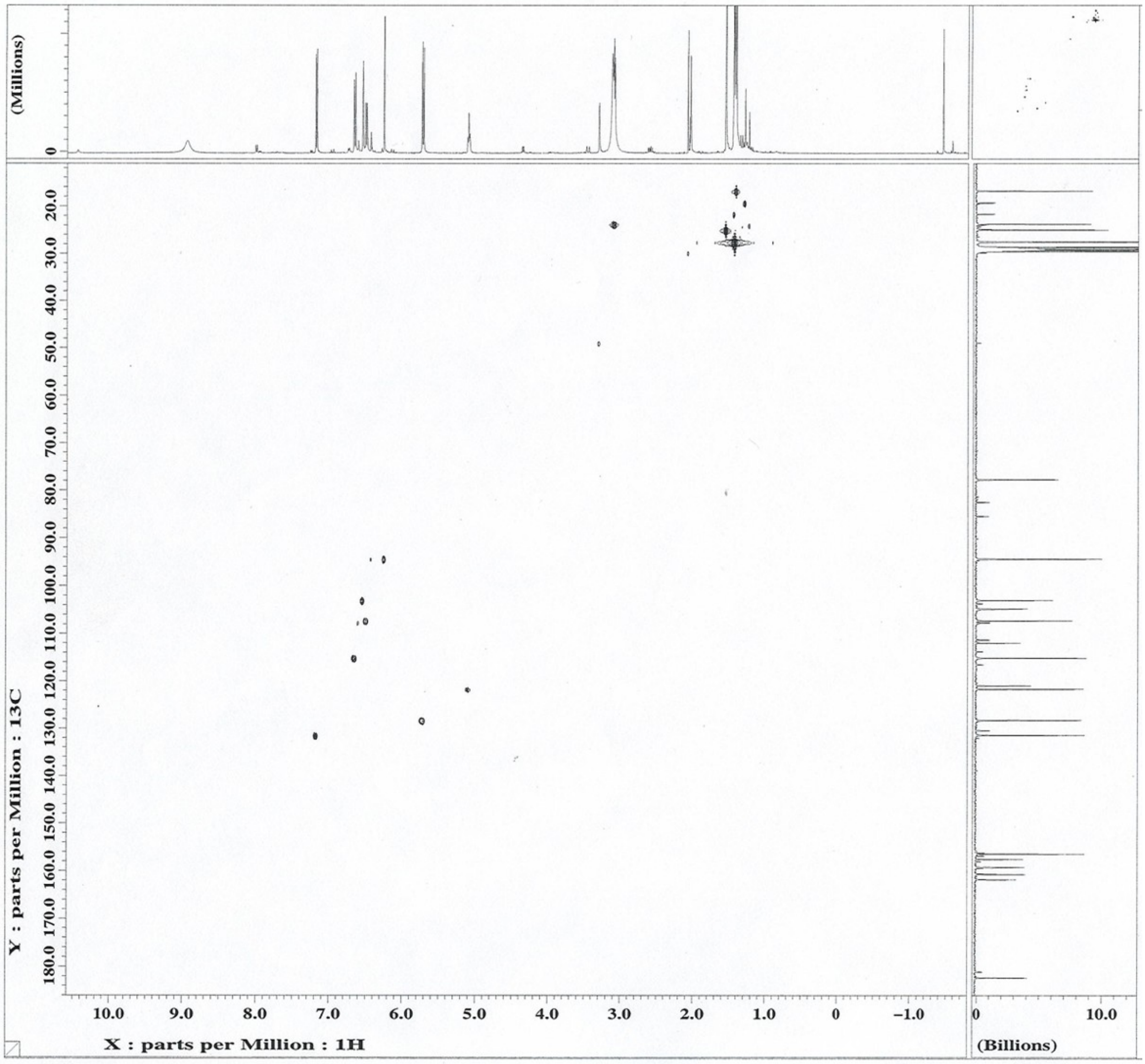

Figure S4. COSY data of cudarflavone B.

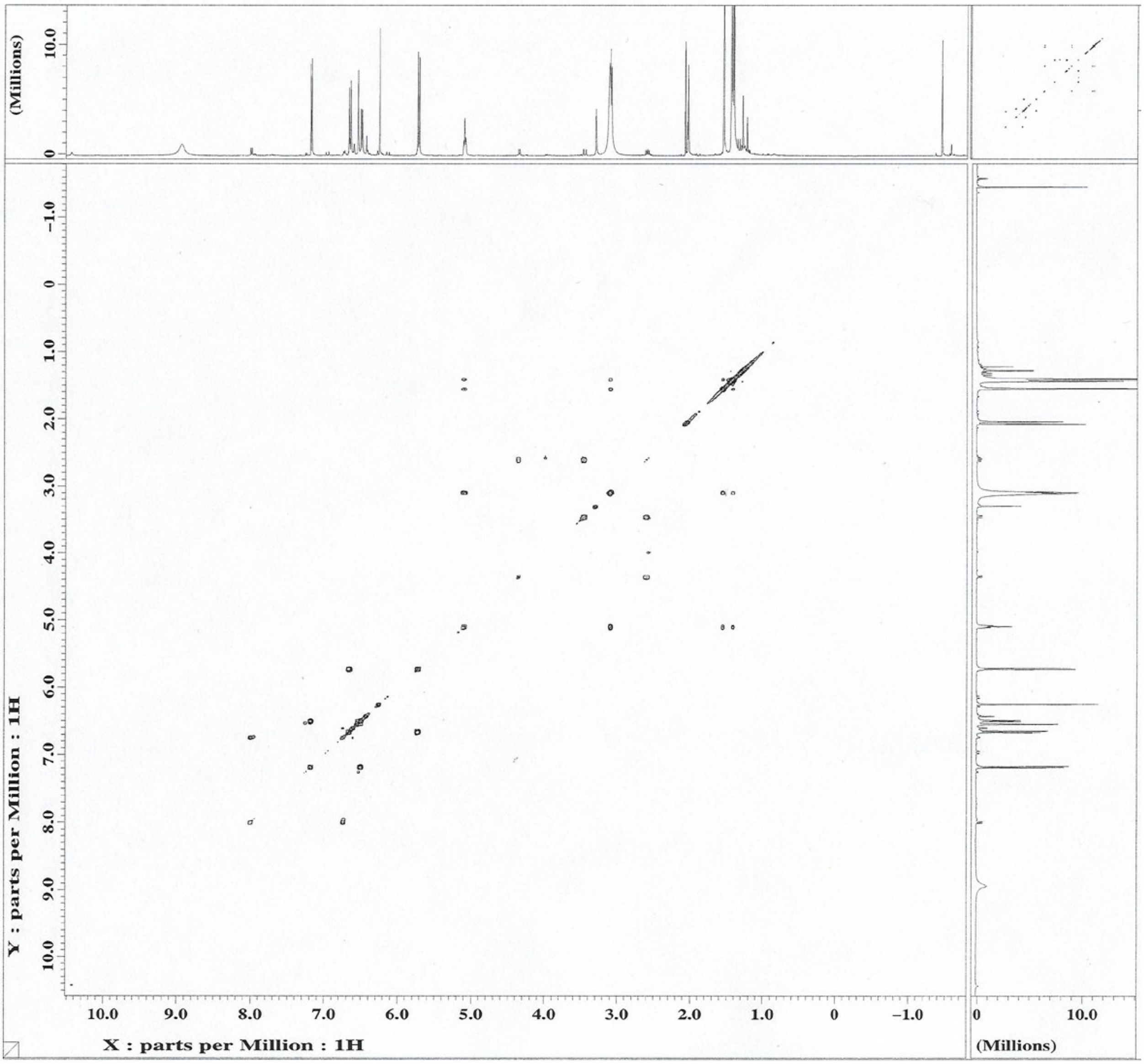

Figure S5. HMBC data of cudarflavone B.

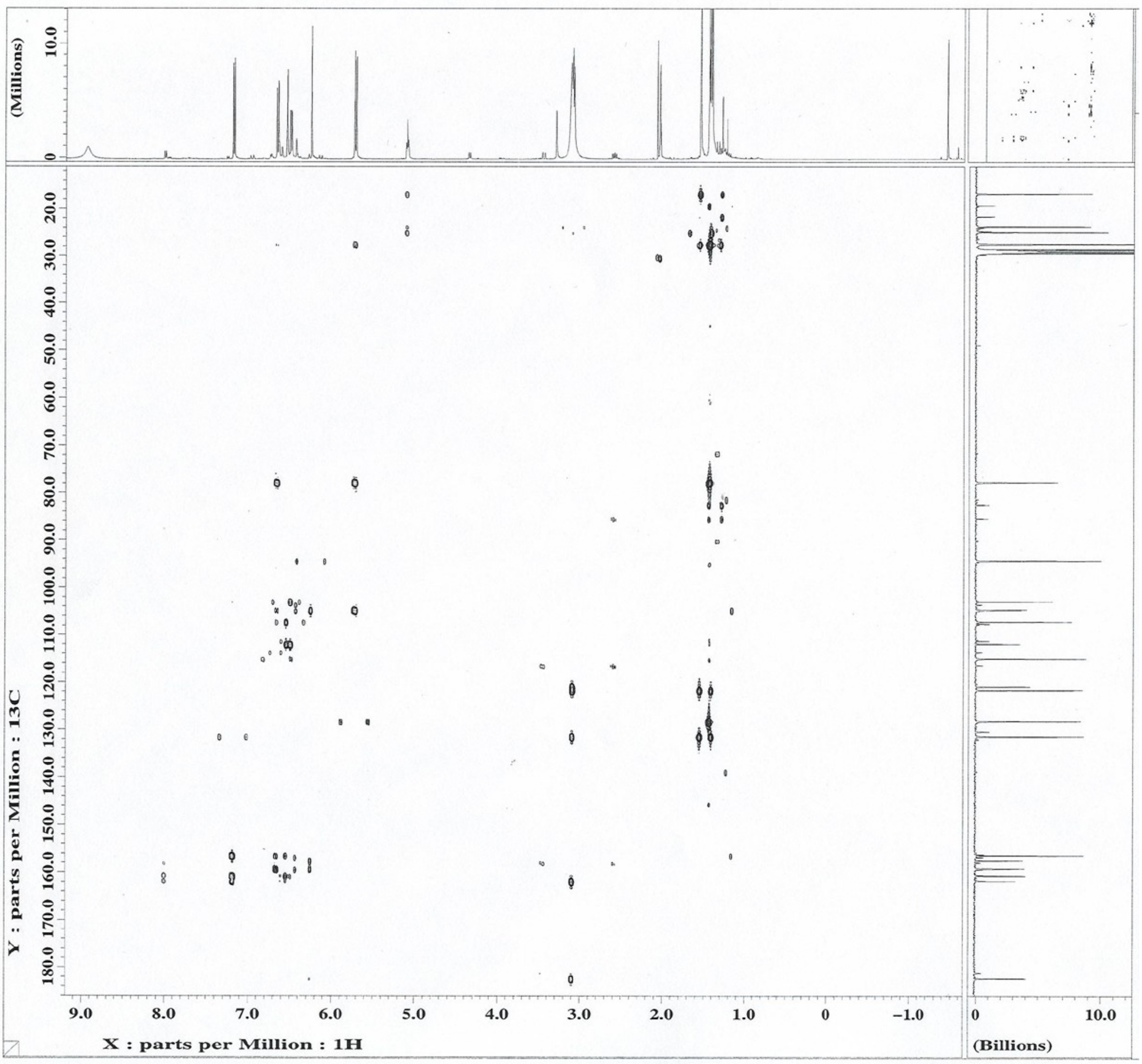

Supplement: Supplementary File 1 [file molecules-19-10818-s001.pdf]
